# Inherited and multiple de novo mutations in autism/developmental delay risk genes suggest a multifactorial model

**DOI:** 10.1186/s13229-018-0247-z

**Published:** 2018-12-13

**Authors:** Hui Guo, Tianyun Wang, Huidan Wu, Min Long, Bradley P. Coe, Honghui Li, Guanglei Xun, Jianjun Ou, Biyuan Chen, Guiqin Duan, Ting Bai, Ningxia Zhao, Yidong Shen, Yun Li, Yazhe Wang, Yu Zhang, Carl Baker, Yanling Liu, Nan Pang, Lian Huang, Lin Han, Xiangbin Jia, Cenying Liu, Hailun Ni, Xinyi Yang, Lu Xia, Jingjing Chen, Lu Shen, Ying Li, Rongjuan Zhao, Wenjing Zhao, Jing Peng, Qian Pan, Zhigao Long, Wei Su, Jieqiong Tan, Xiaogang Du, Xiaoyan Ke, Meiling Yao, Zhengmao Hu, Xiaobing Zou, Jingping Zhao, Raphael A. Bernier, Evan E. Eichler, Kun Xia

**Affiliations:** 1 Center for Medical Genetics, School of Life Sciences, Central South University, Changsha, Hunan China; 2 Department of Genome Sciences, University of Washington School of Medicine, Seattle, WA USA; 3 Mental Health Institute of the Second Xiangya Hospital, Central South University, Changsha, Hunan China; 4 Key Laboratory of Developmental Disorders in Children, Liuzhou Maternity and Child Healthcare Hospital, Liuzhou, Guangxi China; 5 Mental Health Center of Shandong Province, Jinan, Shandong China; 6 Children Development Behavior Center of the Third Affiliated Hospital of Sun Yat-Sen University, Guangzhou, Guangdong China; 7 Center of Children Psychology and Behavior, the Third Affiliated Hospital of Zhengzhou University, Zhengzhou, Henan China; 8 Xi’an Encephalopathy Hospital of Traditional Chinese Medicine, Xi’an, Shaanxi China; 9 Child Mental Health Research Center, Nanjing Brain Hospital Affiliated of Nanjing Medical University, Nanjing, Jiangsu China; 10 Department of Pediatrics, the Xiangya Hospital, Central South University, Changsha, China; 11 Department of Psychiatry, University of Washington, Seattle, WA USA; 12 Howard Hughes Medical Institute, University of Washington, Seattle, WA USA; 13 Key Laboratory of Medical Information Research, Central South University, Changsha, Hunan China; 14 Collaborative Innovation Center for Genetics and Development, Shanghai, China

**Keywords:** Autism spectrum disorders, Targeted sequencing, De novo mutations, Multiple hit, Multifactorial model, Genotype–phenotype relationship

## Abstract

**Background:**

We previously performed targeted sequencing of autism risk genes in probands from the Autism Clinical and Genetic Resources in China (ACGC) (phase I). Here, we expand this analysis to a larger cohort of patients (ACGC phase II) to better understand the prevalence, inheritance, and genotype–phenotype correlations of likely gene-disrupting (LGD) mutations for autism candidate genes originally identified in cohorts of European descent.

**Methods:**

We sequenced 187 autism candidate genes in an additional 784 probands and 85 genes in 599 probands using single-molecule molecular inversion probes. We tested the inheritance of potentially pathogenic mutations, performed a meta-analysis of phase I and phase II data and combined our results with existing exome sequence data to investigate the phenotypes of carrier parents and patients with multiple hits in different autism risk genes.

**Results:**

We validated recurrent, LGD, de novo mutations (DNMs) in 13 genes. We identified a potential novel risk gene (*ZNF292*), one novel gene with recurrent LGD DNMs (*RALGAPB*), as well as genes associated with macrocephaly (*GIGYF2* and *WDFY3*). We identified the transmission of private LGD mutations in genes predominantly associated with DNMs and showed that parental carriers tended to share milder autism-related phenotypes. Patients that carried DNMs in two or more candidate genes show more severe phenotypes.

**Conclusions:**

We identify new risk genes and transmission of deleterious mutations in genes primarily associated with DNMs. The fact that parental carriers show milder phenotypes and patients with multiple hits are more severe supports a multifactorial model of risk.

**Electronic supplementary material:**

The online version of this article (10.1186/s13229-018-0247-z) contains supplementary material, which is available to authorized users.

## Background

In 1943, Leo Kanner first described 11 children with “early infantile autism” as those who manifest “a powerful desire for aloneness” and “an obsessive insistence on persistent sameness” [1]. Since then, the term autism has evolved. Autism spectrum disorder (ASD) is now defined as a group of clinical heterogeneous disorders with deficits in social interaction and repetitive or restrictive behaviors as main clinical characterizations often accompanied by other impairments, such as intelligence and language deficits. Studies conducted on several continents (Asia, Europe, and North America) indicate a prevalence of approximately 0.12–2.64% [2–6].

Changes in diagnostic criteria have been accompanied by large-scale, genome-wide, and targeted sequencing analyses dramatically accelerating the discovery of candidate genes associated with ASD [7–12]. Detailed phenotype–genotype correlations have been established for several high-risk genes in the last several years, such as *CHD8* [13], *ADNP* [14], *DYRK1A* [15], and *POGZ* [16], emphasizing both the clinical and genetic heterogeneity of ASD. Despite these advances, several limitations remain. Only a small fraction of the genetic risk has been defined, the penetrance of most mutated genes is unknown, and genotype–phenotype correlations in most cases are unclear.

Previously, we presented results from targeted sequencing of 1543 ASD probands from the Autism Clinical and Genetic Resources in China (ACGC) [17]. This study extends the cohort to 2926 probands (~ 2000 from trios or quads) with the aim to identify novel autism risk mutations, their associated phenotypes, and the transmission characteristics of potentially pathogenic mutations within families. In addition to discovering potential novel ASD risk loci in the Chinese population, we report the discovery of patients with de novo mutation (DNM) in more than one autism risk gene, suggesting that multiple genes may contribute to ASD pathology. Consistent with this model, we identify families with inherited likely gene-disrupting (LGD) mutations in genes primarily associated with DNM emphasizing the importance of clinical evaluation and follow-up genetic counseling.

## Methods

### Study samples

Study samples were selected from the ACGC [17]. This collection includes seven clinical referring centers (Additional file 1: Figure S1) and consists of ~ 10,000 ASD familial DNA samples. Patients were diagnosed primarily according to DSM-IV/V criteria documenting additional comorbid conditions where possible. Of the 3120 ASD probands in the ACGC, 2276 represent complete parent–child trios or quads and the majority are simplex autism with no family history of ASD. Peripheral-blood DNA of all individuals with ASD and their parents, if available, was collected with informed consent by seven coordinating centers. Genomic DNA was extracted from the whole blood. All study procedures were in accordance with the ethical standards of the Institutional Review Board of the School of Life Sciences at Central South University, Changsha, Hunan, China.

### Targeted sequencing of the ACGC

Previously, we targeted 213 candidate genes for sequencing in 1647 probands (1543 probands after QC) (phase I), as previously described [17] (Table 1). We define recurrent mutations based on the presence of independent mutations in the same gene but not necessarily at the same site. The candidate gene set consisted of recurrent DNM calls (including LGD and missense) identified from exome sequencing of autism families—primarily the Simons Simplex Collection (SSC) and the Autism Sequencing Consortium (ASC). In addition, we also included genes with recurrent de novo (DN) events among intellectual disability (ID) and developmental delay (DD) probands and genes disrupted by copy number variants (CNVs) but for which no DN single-nucleotide variant has yet been identified.

**Table 1:** Summary of sample numbers in ACGC targeted sequencing

Categories	Phase I	Phase II			Total
		Stage 1	Stage 2	Total	
Probands subjected to sequencing	1647 (1086)	851 (735)	622 (455)	1473 (1190)	3120 (2276)
Probands after QC	1543 (1045)	784 (672)	599 (437)	1383 (1109)	2926 (2154)
Targeted gene numbers	211	211	85	–	–
Targeted gene numbers after QC	187	187	85	–	–

Numbers in parentheses indicate the probands with both parents’ DNA available

In this study, we sequenced an additional 1473 probands (Table 1) from the ACGC using single-molecule molecular inversion probes (smMIPs) [7, 18]. The samples were sequenced and analyzed using a staged approach. During the first stage, we combined the results from phase I sequence data from an additional 851 probands for 211 genes from the original study. During the second stage, we reduced the gene set and focused on targeted resequencing of 85 genes selected from the 211 genes according to the latest progress and our in-house data for the remaining 622 probands. We combined the smMIP data and quality-controlled data from both phase I and phase II for 3120 total patients. After controlling for coverage and uniformity, data from 2926 probands (phase II, 1383 probands) with 2154 from trios or quads (phase II, 1109 probands) passed quality control (QC) (Table 1, Additional file 2: Data 1, Additional files 3 and 4: Figures S2 and S3). All subsequent analyses are based only on those genes and samples that passed the following QC metrics.

### Quality control and variant calling

We applied a similar pipeline as in phase I for QC and variant calling. For QC, only the data from the individuals with more than 75% of the target (with coverage over 8×) and genes with more than 30% of the individuals (and a minimum of eightfold sequence coverage) were used for the following analysis. Sequencing was performed using the Illumina HiSeq2000 platform. Reads were aligned against hg19 with BWA-MEM (v0.7.13) [19] after removing incorrect read pairs and low-quality reads; single-nucleotide variants and indels were called with FreeBayes (v0.9.14). We consider variants exceeding eightfold sequence coverage and read quality over 20 (QUAL > 20) for annotation with SeattleSeq, as previously described [20]. Variants with allele count (AC) ≤ 3 (0.1%) and allele frequency < 0.1% in the Exome Aggregation Consortium (ExAC) database were considered for subsequent analysis and validation.

### Variant validation and microsatellite analysis

We validated variants by PCR amplification (500 bp amplicons) followed by Sanger sequencing. We tested transmission for all validated variants wherever parental DNA was available. To eliminate nonpaternity, we performed an independent microsatellite analysis using 8–13 autosomal microsatellite markers for each family. Microsatellite loci were amplified by PCR using fluorescently labeled primers, and labeled products were analyzed by capillary electrophoresis using GeneMarker software and the ABI 3730XL DNA Analyzer.

### Statistical analyses

We applied two statistical models to assess the excess of DNMs for the 187 genes that passed QC. The first, a chimpanzee–human divergence (CH) model [7], considers the length of the gene and divergence between chimpanzees and humans to predict the expected number of DNMs, while the model denovolyzeR [21] estimates mutation rates based on trinucleotide context and accommodates known mutational biases such as CpG hotspots. Default parameters were used for both models with an expected rate of 1.5 DNMs per exome for the CH model [7]. *P* values in the ACGC-only analysis were corrected for 187 genes. *P* values in the ACGC, SSC, and ASC combined meta-analysis were corrected for 18,945 genes for which mutation rates have been estimated. A comparison of Combined Annotation Dependent Depletion (CADD) score distribution between missense DNMs within *SCN2A* and *CHD8* from ASD patients and rare missense mutations within *SCN2A* and *CHD8* from ExAC controls was performed using the Wilcoxon signed-rank test. The relationship between affected status and the number of DNMs in the SSC exome data was tested using a logistic regression model with affected status as a response variable and DNM numbers, father’s birth age, and gender as predictor variables. This model took the form: *logit[P(affectedStatus)] ~ DNM Number + FatherAgeAtBirth + Gender*. Affected status represents a binary classification (proband (*n* = 2508) and sibling (*n* = 1911)) for each individual from the SSC whole-exome sequencing study cohort [9]. The DNM number represents the number of DNMs in each individual that result in an autosomal amino-acid alteration (LGD, missense, and in-frame indels). FatherAgeAtBirth is defined as the father’s age in months at the time of the child’s birth. The model was run with the glm function in the R statistical package for all individuals. Subsequently, males and females were separately tested using the same model without gender as a co-variable. The relationship between Social Responsiveness Scale (SRS) score and DNM number was tested using linear regression models with SRS score as the response variable, DNM number as the predictor variable, and gender as a co-variable. The relationship between nonverbal IQ (NVIQ) and DNM number was tested using the same model as for the SRS score. The relationship between seizures and DNM number was tested using the logistic regression model with seizure status (yes/no) as the response variable, DNM number as the predictor variable, and gender as a covariant. *P* values of these three phenotype analyses were corrected with false discovery rate (FDR) approach.

## Results

### Mutation discovery

We discovered 2496 rare variants that are predicted to alter the amino acid sequence or disrupt a gene in phase II samples. We selected LGD mutations (*n* = 99) and missense mutations with a CADD score of equal to or greater than 30 (termed MIS30+; *n* = 133), because of their higher DN and pathogenic probability [17, 22], for validation. In total, we validated 221 putative severe mutations (92 LGD and 129 MIS30+) yielding an overall validation rate of 95.3% (Additional file 5: Data 2). Where parental DNA was available, we assessed the inheritance status. In order to identify potential cases of nonpaternity, we assessed the paternal transmission of rare single-nucleotide variants or microsatellite analysis. We also confirmed the inheritance of all rare, less severe, missense mutations (CADD < 30, termed MIS30-) for genes (*n* = 38) where severe DNMs had been identified (LGD and MIS30+) in the ACGC and confirmed 3% (20/630) as DN (Additional file 6: Data 3). In total, we validated 104 DNMs (55 in phase II), including 60 LGD (31 in phase II), 8 MIS30+ (4 in phase II) and 36 MIS30- (20 in phase II) mutations (Additional file 7: Data 4). DNMs in these 38 genes account for 4.83% of all QC-passing ACGC patients. The proportion of all kinds of DNMs in phase II was consistent with the proportion observed in phase I.

### Recurrent new mutations and candidate genes

We calculated the overall probability of detecting 60 or more LGD DNMs in 187 QC-passed genes using the CH model by setting an expected rate of 1.5 DNMs per exome as *q* = 1.35 × 10^−38^ (two-tailed binomial test) with an odds ratio (OR) of 11.6 (95% confidence interval 8.9–14.8). For the known autism risk genes confirmed in previous studies, *SCN2A* is still the most frequently mutated in this study. We identified a total of 24 families with *SCN2A* DNMs accounting for 1.1% of all patients (Additional file 8: Figure S4). Missense DNMs in *SCN2A* map predominantly to the ion transport domain (Additional file 9: Figure S5a), consistent with sodium ion transport dysfunction in the synapse [23]. After combining the reported DNMs identified in ASD patients, three recurrent missense DN amino acid sites were identified at R937 (4), R379 (2), and G1744 (2) (Additional file 9: Figure S5a).

*CHD8* is the second most frequently mutated gene in this cohort. We found five LGD and two missense DNMs (Additional file 8: Figure S4) consistent with a significant excess of truncating mutations in ASD cohorts. Combining data from previous exome or targeted capture sequencing studies [8–10], we report an excess of missense DNMs (*n* = 8) in *CHD8* by the CH model (*p* = 9.96 × 10^−8^, *q* = 0.002, OR = 15.03). One recurrent missense DN amino acid site was identified at M904 (2) with three missense DNMs mapping to the DEXDc domain (Additional file 9: Figure S5b). Overall, CADD score distributions of the missense DNMs within *SCN2A* and *CHD8* from ASD patients are significantly higher than the CADD distributions of rare missense mutations within *SCN2A* (*p* = 2 × 10^− 4^) and *CHD8* (*p* = 1.8 × 10^− 3^) from ExAC samples (Additional file 9: Figure S5c). After *SCN2A* and *CHD8*, *MECP2* (3 LGD, 4 missense), *ASXL3* (3 LGD, 2 missense), *DYRK1A* (3 LGD, 1 missense), and *WDFY3* (1 LGD, 3 missense) are the top frequently mutated genes (Additional file 10: Figure S6). In total, we observe recurrent LGD DNMs in 13 genes, namely *SCN2A*, *CHD8*, *MECP2*, *ASXL3*, *DYRK1A*, *DSCAM*, *WAC*, *FOXP1*, *MED13L*, *CTTNBP2*, *ZNF292*, *TNRC6B*, and *CDKL5* (Table 2).

**Table 2:** Gene-specific significance in the ACGC cohort and in the ACGC+SSC+ASC combined samples based on two statistical models

Gene	ACGC (*n* = 2145)										ACGC+SSC+ASC (*n* = 6107)						
	Phase-I		Phase-II		All		CH model		denovolyzeR		LGD	MIS	All	CH model		denovolyzeR	
	LGD	MIS	LGD	MIS	LGD	MIS	LGD_q	prot_q	LGD_q	prot_q				LGD_q	prot_q	LGD_q	prot_q
*SCN2A* ^*†*^	6	4	5	9	11	13	*3.68E−25*	*1.23E−40*	*4.21E−18*	*1.05E−29*	15	21	36	*7.77E−28*	*2.23E−49*	*3.52E-22*	*3.35E-42*
*CHD8* ^*†*^	3	1	2	1	5	2	*3.18E−09*	*7.56E−08*	*5.40E−07*	*1.68E−04*	14	4	18	*7.49E−25*	*7.45E−18*	*3.52E−22*	*1.19E−13*
*MECP2* ^*†*^	1	3	2	1	3	4	*1.00E−06*	*1.12E−11*	*1.78E−05*	*4.12E−08*	3	5	8	*6.26E−04*	*1.33E−08*	*1.78E−03*	*1.42E−06*
*DYRK1A* ^*†*^	1	1	2	0	3	1	*1.51E−05*	*2.78E−04*	*5.92E−05*	*2.75E−03*	8	1	9	*3.78E−13*	*2.51E−07*	*1.31E−13*	*4.23E−07*
*ASXL3* ^*†*^	0	1	3	1	3	2	*7.28E−04*	*5.62E−03*	*1.82E−04*	*4.29E−03*	5	2	7	*6.33E−04*	0.894	*6.03E−06*	*0.0200*
*WAC* ^*†*^	0	0	2	0	2	0	*9.05E−04*	*0.0311*	*5.14E−03*	0.2322	4	0	4	*2.12E−05*	0.0532	*6.57E−05*	0.1384
*FOXP1* ^*†*^	0	0	2	1	2	1	*1.04E−03*	*3.46E−03*	*0.0103*	*0.0285*	4	1	5	*3.36E−05*	*0.014*	*3.81E−04*	*0.0235*
*CTTNBP2*	0	1	2	0	2	1	*4.69E−03*	*0.0189*	*0.0226*	0.2162	3	1	4	*0.0331*	1	0.1072	1
*TNRC6B* ^*†*^	0	0	2	0	2	0	*7.00E−03*	0.2549	*0.0158*	0.5818	4	0	4	*1.97E−03*	1	*1.17E−03*	1
*MED13L* ^*†*^	1	0	1	0	2	0	*7.00E−03*	0.2549	*0.0386*	0.9485	4	1	5	*1.97E−03*	0.9197	*9.40E−03*	1
*CDKL5* ^*†*^	1	0	1	0	2	0	*8.26E−03*	0.2732	*7.14E−03*	0.3409	2	1	3	1	1	0.5527	1
*DSCAM* ^*†*^	2	0	0	1	2	1	*0.0130*	0.0770	*0.0226*	0.3035	6	2	8	*1.03E−05*	*0.0272*	*1.55E−06*	*0.0135*
*ZNF292*	1	0	1	0	2	0	*0.0139*	0.6051	*0.0158*	0.8299	3	2	5	0.2763	1	0.0508	1
*POGZ* ^*†*^	1	1	0	0	1	1	0.0507	*0.0153*	0.3864	0.5818	4	4	8	*2.40E−06*	*1.84E−09*	*3.48E−03*	*7.13E−04*
*WDFY3*	1	1	0	2	1	3	0.2966	*0.0153*	0.7292	0.2736	3	7	10	0.2778	*7.21E−04*	1	*5.44E−03*
*NAA15* ^*†*^	0	0	1	1	1	1	0.0851	*0.0228*	0.3452	0.3025	2	2	4	0.1747	*0.0287*	1	0.4185
*CUL3*	1	0	0	1	1	1	0.1056	*0.0446*	0.3825	0.3035	2	1	3	0.2778	1	1	1
*STXBP1* ^*†*^	1	0	0	0	1	0	0.1056	0.6051	0.3267	1	1	1	2	1	1	1	1
*ADNP* ^*†*^	1	0	0	0	1	0	0.1314	0.9222	0.2294	1	5	0	5	*2.40E−06*	0.0764	*4.55E−07*	0.0669
*DDX3X* ^*†*^	0	1	1	0	1	1	0.1335	0.0871	0.2367	0.2989	2	1	3	0.7186	1	0.4989	1
*RALGAPB*	0	0	1	1	1	1	0.1335	0.0815	0.3267	0.5816	2	1	3	0.6237	1	1	1
*GIGYF2*	1	1	0	0	1	1	0.1335	0.0770	0.6428	0.5818	2	3	5	0.7186	*0.0363*	1	0.5211
*NCKAP1*	1	0	0	0	1	0	0.1335	0.9135	0.5383	1	3	0	3	*0.0206*	1	0.4100	1
*GRIN2B* ^*†*^	1	0	0	0	1	0	0.1359	1	0.2367	1	4	0	4	*4.61E−04*	1	*1.17E−04*	1
*MYT1L* ^*†*^	1	0	0	0	1	0	0.1717	1	0.2294	1	2	2	4	1	1	0.4100	1
*SHANK2*	0	0	1	0	1	0	0.2017	1	0.3267	1	2	1	3	1	1	1	1
*SYNGAP1* ^*†*^	1	0	0	0	1	0	0.2966	1	0.3154	1	6	3	9	*1.84E−05*	*0.0168*	*8.04E−08*	*8.79E−05*
*PHIP* ^*†*^	1	0	0	0	1	0	0.2966	1	0.5095	1	1	1	2	1	1	1	1
*CHD2* ^*†*^	0	0	1	1	1	1	0.3536	0.9217	0.5095	0.7736	4	4	8	*0.0331*	0.5391	*0.0125*	*3.31E−03*
*SHANK1*	1	0	0	0	1	0	0.3536	1	0.3267	1	1	1	2	1	1	1	1
*DOCK8*	1	0	0	0	1	0	0.4837	1	0.3267	1	2	0	2	1	1	1	1
*ANK2*	0	1	1	0	1	1	0.7356	1	0.6639	1	5	4	9	0.0587	1	*2.54E−03*	*0.0396*
*ASH1L* ^*†*^	0	1	0	1	0	2	1	0.2732	1	1	3	2	5	0.0825	1	0.5350	1
*RIMS1*	0	0	0	1	0	1	1	1	1	1	2	1	3	0.7186	1	1	1
*TSC2* ^*†*^	0	1	0	0	0	1	1	1	1	1	0	4	4	1	1	1	1
*KMT2C*	0	1	0	1	0	2	1	1	1	1	1	4	5	1	1	1	1
*ITPR1*	0	1	0	0	0	1	1	1	1	1	0	3	3	1	1	1	1
*WHSC1*	0	0	0	1	0	1	1	0.9499	1	1	1	1	2	1	1	1	1

Significant *q* values (< 0.05) are italicized

*LGD* likely gene-disrupting variants, *MIS* missense variants, *LGD_q q* values of truncated DNMs (FDR corrected), *prot_q q* values of LGD and missense DNMs (FDR corrected)

^†^Previously well-defined genes in neurodevelopmental disorders

**Fig. 1:**
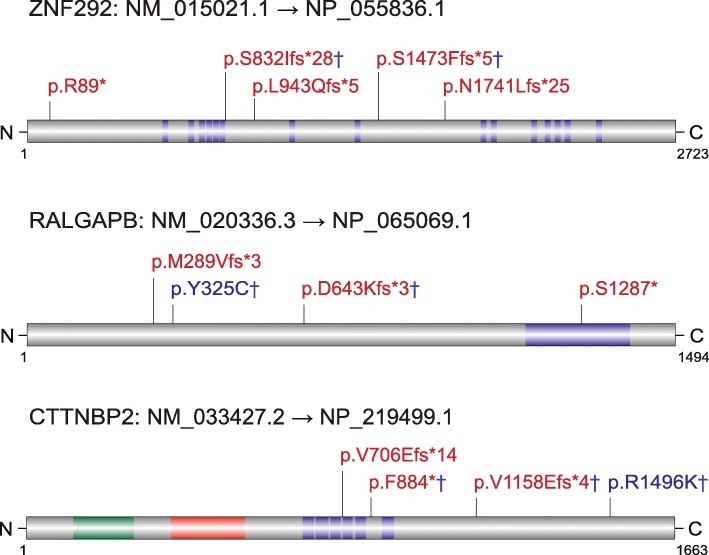
Distribution of DNMs in two potential novel autism risk genes (*ZNF292*, *RALGAPB*) and *CTTNBP2*. Dagger symbol indicates the DNMs reported in this study. *ZNF292*: p.R89* was reported in an ASC patient, p.L943Qfs*5 was reported in an ID patient, p. N1741Lfs*25 was reported in DDD study. *RALGAPB*: p.M289Vfs*3 was reported in an ASC patient, p.S1287* was reported in an EPI4K patient. *CTTNBP2*: p.V706Efs*14 was reported in an SSC patient

We applied two statistical models (see the “Methods” section) to assess the probability of excess of DNMs for the 187 genes. We identified 17 genes that reached significance for an excess of DNMs by the CH model and 13 genes by denovolyzeR (*q* < 0.05) in the ACGC cohort (Table 2). Combining the ACGC analysis and ACGC-SSC-ASC analysis, 21 total genes reached significance by the CH model and 18 genes reached significance by denovolyzeR. *ZNF292*, which has not been reported as significant in previous studies, was implicated as a novel autism risk gene in the ACGC cohort by both models (*q* = 0.014, CH model; *q* = 0.016, denovolyzeR model) (Table 2). We note that one LGD DNM was recently reported in the DDD study [24] and another LGD DNM was reported in an ID patient [25] (Fig. 1) clearly implicating this gene in ID as well as autism. In addition, we established recurrent LGD DNMs for *RALGAPB* by combining SSC and ASC exome data, although it is still not significant (*q* = 0.13, CH model; *q* = 0.33, denovolyzeR model) (Fig. 1, Table 2). Interestingly, an LGD DNM was reported in an epilepsy patient from the EPI4K study [26] (Fig. 1). *CTNNBP2* was previously implicated for autism risk by the TADA (Transmission And De novo Association) test [11], and we now report DN significance based on the discovery of one missense and two LGD DNMs (*q* = 0.005, CH model; *q* = 0.02, denovolyzeR model) (Fig. 1, Table 2). We did not observe other potential pathogenic mutations in other ASD risk genes sequenced in this study in the patients with *ZNF292*, *RALGAPB*, and *CTNNBP2* DNMs.

### Clinical evaluation of ASD-relevant mutations

For patients carrying DNMs in autism risk genes, we reviewed the clinical details and made an attempt wherever possible to recontact families in order to assess phenotype, perform a physical examination, and assess co-occurring conditions. We observed significant ID, DD, and other comorbidities, such as behavior problems, in the well-defined or syndromic ASD genes—*SCN2A*, *MECP2*, *FOXP1*, *ADNP*, and *ASXL3*—which is consistent with the previous genotype–phenotype correlation analysis (Additional file 11: Data 5). Since we detected a relatively large number of probands with DNMs in *SCN2A*, which raises the possibility of dominant-negative or gain-of-function effects of the missense DNMs, we compared the phenotypes between patients with LGD DNMs (*n* = 6) and patients with missense DNMs (*n* = 11). However, we did not observe a significant difference between the two groups (Additional file 11: Data 5). While the number of patients with recurrent mutations in the candidate genes is too few to make definitive genotype–phenotype correlations with specific genes, several interesting trends were observed. First, the majority of patients with severe DNMs and a cognitive assessment showed evidence of some form of intellectual impairment. Only *TNRC6B*, *NCKAP1*, and one of the two *ZNF292* LGD DNMs occur in autism patients with an IQ in the normal range.

Since microcephaly and macrocephaly have long been recognized as a co-occurring condition of ASD, we also assessed patients for abnormalities in head circumference (HC). In addition to *CHD8*, patients with LGD mutations within *WDFY3*, *KMT5B*, and *GIGYF2* have notably larger HC. Three patients with LGD mutations in *WDFY3* (1 DN, 1 inherited, and 1 undetermined inheritance) were identified with HC *Z*-scores of 2.5, 3.0, and 2.8, respectively. Two inherited LGD mutations were identified in *KMT5B* in two ACGC patients and both showed evidence of macrocephaly. Similarly, two *GIGYF2* LGD mutations (1 DN and 1 undetermined) were identified in two patients and both were macrocephalic. In contrast, patients with *DYRK1A*, *CDKL5*, and *MED13L* LGD mutations have smaller HC, consistent with previous reports [15, 27].

### Multiple DNMs in ASD patients

**Fig. 2:**
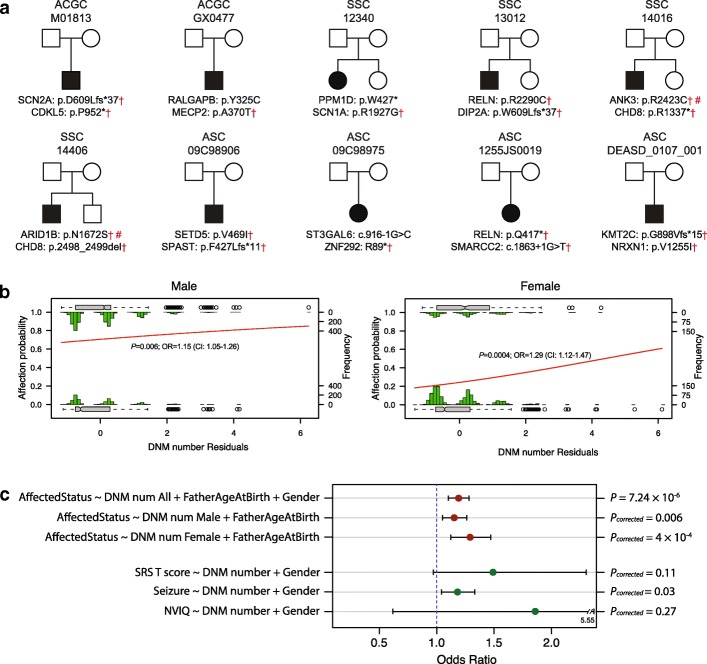
Multiple-hit model for ASD. **a** Ten families and corresponding double-hit DNMs in the ACGC, SSC and ASC cohorts are shown. Dagger symbol indicates the genes listed as autism risk genes in SFARI; number sign indicates the variants presented in ExAC database. **b** The logistic histogram plot shows that both males and females have a higher probability of being affected with an increase in the number of DNMs even after correcting for paternal age effect. Females show a higher odds ratio (OR) than males for this additional DNM effect. **c** The plot shows the distribution of OR and the corresponding 95% CI of regression models, which predict affected status and different phenotype severity by DNM numbers

During our analysis of the ACGC cohort, we identified two patients with two LGD DNMs in genes where each individually had reached significance for an excess for DNMs (Fig. 2a). Most notably, patient M01813 carried LGD DNMs in autism risk genes *SCN2A* and *CDKL5*, albeit the latter occurs near the terminal portion of the protein*.* The other patient, GX0477.p1, carried missense DNMs from *MECP2* and *RALGAPB*. We assessed the frequency of such “double-hit” DNMs for our initial target set of 187 genes in both the SSC and ASC exome sequence datasets. We identified four additional SSC probands and four ASC probands with double-hit DNMs of which 6/8 pairs of genes were also classified as autism risk genes in the Simons Foundation Autism Research Initiative (SFARI) Gene database (Fig. 2a). Of those, only two missense mutations were presented in the ExAC database, although variants identified in ExAC do not indicate they are benign [28]. No such double-hits were identified in the unaffected SSC siblings. Although multiple-hit events are expected to occur more frequently in probands than siblings, we further investigated their effect on phenotype and gender differences among probands.

Using the SSC exome datasets, we examined the relationship between affected status and the number of DNMs and correcting for father’s age at birth and gender (see the “Methods” section). Individuals with increasing DNM numbers are more likely to be affected (*p* = 7.24 × 10^−6^, OR = 1.19) (Additional file 12: Figure S7). We further analyzed male and female samples separately by the same analysis. Both male (*p* = 0.006, OR = 1.15) and female (*p* = 0.0005, OR = 1.29) probands demonstrate a significant relationship between affected status and the number of DNMs (Fig. 2b), even after correcting for father’s age at birth. Interestingly, we observed that, compared to male samples, females demonstrated an increased odds ratio for additional DNMs. This result is consistent with the “female protective model,” where female probands may require additional mutational burden to reach a clinical diagnosis of autism [29]. We repeated the same analyses removing cases with no DNM under the same regression models. We observed that individuals with increased DNMs are more likely to be affected (*p* = 0.028, OR = 1.15) (Additional file 13: Figure S8a). When samples are stratified by genetic sex, we observe a slight increase but no significant effect among males (*p* = 0.6, OR = 1.09; *p* values corrected for two tests) (Additional file 13: Figure S8b), while females demonstrate a stronger (*p* = 0.037, OR = 1.3) effect than the grouped analysis (Additional file 13: Figure S8c). These associations should be regarded as suggestive until replicated with cohorts of large sample size.

Finally, to test whether patients with more DNMs demonstrate more severe phenotypes, we explored the relationship between three autism-related phenotypes (autism symptom impairment, seizure, and NVIQ) and DNM numbers. Autistic severity, per parent report on the SRS [30], increased with increasing DNM numbers with marginal significance (*p* = 0.07, *q* = 0.11, OR = 1.49; Fig. 2c). Similarly, there is also a significant trend for increasing frequency of seizures (*p* = 0.01, *q* = 0.03, OR = 1.18; Fig. 2c) with the increase of DNMs. While patients with increased DNMs appear to have decreased IQ, this trend is not significant (*p* = 0.27; Fig. 2c).

### Inheritance of potential high-risk mutations

**Fig. 3:**
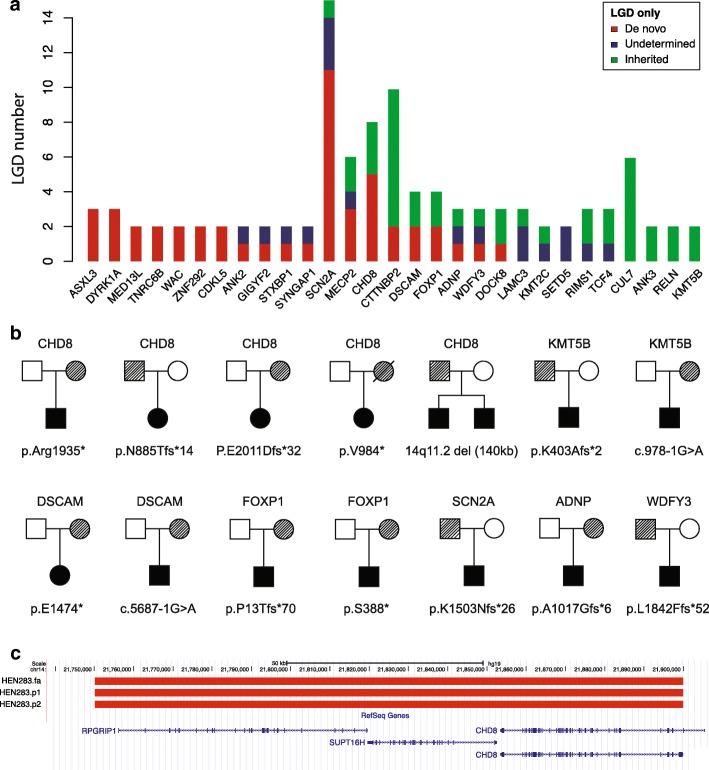
Inheritance of high-risk ASD genes. **a** The number of inherited and DNMs by autism risk gene within the ACGC. **b** Families with inherited LGD mutations or gene-disrupting CNVs in ASD high-risk genes, including *CHD8* (5), *KMT5B* (2), *DSCAM* (2), *FOXP1* (2), *SCN2A* (1), *ADNP* (1), and *WDFY3* (1). **c** Genomic location of inherited CNV-disrupting *CHD8* within an ASD family from the ACGC cohort. LGD, likely gene-disrupting

Although this study focused primarily on DNMs, we also identified 40 LGD mutations within known autism genes where transmission was observed from supposed unaffected parents to ASD offspring (22 maternal, 18 paternal) (Fig. 3a). Specifically, we identified 12 inherited LGD mutations in genes where a burden of excess DNMs had been previously described, including *CHD8* (3), *KMT5B* (2), *DSCAM* (2), *FOXP1* (2), *SCN2A* (1), *ADNP* (1), and *WDFY3* (1) (Fig. 3b). Similarly, we also discovered a CNV disrupting *CHD8* through our clinical work. The 140 kbp deletion was transmitted from a father to both affected siblings and is absent from the Database of Genomic Variants. The deletion was further validated by array comparative genomic hybridization (Fig. 3c). Combined with a previously reported inherited LGD [13], we report five ASD families with inherited *CHD8* LGD mutations (Fig. 3b).

To evaluate the ASD phenotypes of parents carrying potential pathogenic LGD mutations, we attempted to recontact all ACGC families carrying inherited *CHD8* and *KMT5B* LGD mutations for clinical reevaluation. Wherever possible, we assessed IQ using the age-appropriate Wechsler battery, HC, and autism-related traits using the Broad Autism Phenotype Questionnaire (BAPQ). Three families with inherited LGD mutation of *CHD8* (as well as one family with inherited LGD mutation of *KMT5B*) were successfully recontacted. All three parents (two mothers and one father) carrying *CHD8* LGD mutations show lower NVIQ scores (< 80), which fall in the borderline range (Table 3). Their scores, however, are significantly higher than their affected children, who are generally severely impaired (IQ < 40), and in one case, cognitive deficits were so severe, an estimate of IQ could not be determined. Two of the children show increased HC consistent with the *CHD8* phenotype [13], although only one carrier mother could be clinically classified as macrocephalic. Macrocephaly was also observed for the father carrying the 140 kbp deletion of *CHD8* (*Z*-score = 5.5). BAPQ data for all three *CHD8* carriers suggests that parents carrying *CHD8* LGD mutations (Additional file 14: Figure S9) show autistic traits with high scores across the domains of autism: behavior (rigid personality) and social communication (pragmatic language deficits). Similar to *CHD8*, the single parent carrying *KMT5B* LGD mutations also shows a lower IQ within normal range and features consistent with a broader autism phenotype. The data clearly argue that even among genes where there is strong evidence of increased DNM burden, an LGD mutation may not be necessary or sufficient to develop autism suggesting reduced penetrance or variability of expressivity. Overall, these data support the idea that instead of non-penetrance, the mutations are resulting in variable phenotypes consistent with a range of ASD manifestations.

**Table 3:** IQ, BAPQ, and physical examination information of parents with *CHD8* LGD mutations and the affected offspring

Sample ID	Mutation	Sex	Age	WAIS/WISC			BAPQ				HC (*Z*-score)	Height	Weight	BMI
				Verbal	Nonverbal	Full-scale	Aloof	Pragmatic	Rigid	Overall				
GX0347.p1	p.R1935*	M	62 M	31	26	21	–	–	–	–	52.5(1.15)	126.2	25	15.6
GX0347.mo	p.R1935*	F	29 Y	88	75	80	3	2.79	3.54	3.11	57.3(2.48)	161.7	67.5	25.8
GX0540.p1	p.N855Tfs*14	F	163 M	–	–	36	–	–	–	–	58.5(3.58)	161.5	84	32.2
GX0540.fa	p.N855Tfs*14	M	42 Y	95	79	87	2.88	3.21	3.88	3.32	58.7(1.53)	167.8	71	25.22
HN0277.p1	p.E2011Dfs*32	F	59 M	–	–	–	–	–	–	–	53(2.26)	112	20	15.9
HN0277.mo	p.E2011Dfs*32	F	26 Y	88	75	80	2.79	2.88	3.92	3.19	–	–	–	–

*M* months, *Y* years, *WAIS* Wechsler Adult Intelligence Scale, *WISC* Wechsler Intelligence Scale for Children, *BAPQ* Broad Autism Phenotype Questionnaire

## Discussion

We have sequenced the protein-encoding region of 187 ASD candidate genes in 784 autism patients and 85 genes in 599 additional autism patients and performed a meta-analysis from 2926 ACGC patients to identify novel risk genes, mutations, and genotype–phenotype relationships. Severe DNMs in *SCN2A* and *CHD8* (including missense burden) account for 1.5% of the ACGC cohort with an additional 3.33% of the patients showing DNMs in an additional 36 genes, most of which reach DN significance. Patients with recurrent *WDFY3*, *GIGYF2*, and *KMT5B* LGD mutations show evidence of increased HC size, implicating novel macrocephaly-associated ASD genes. Consistent with this observation, it has been reported that loss of Wdfy3 in mouse leads to regional enlargements of the cerebral cortex [31].

*ZNF292* was implicated as a novel ASD risk gene in this study. *ZNF292* encodes a KRAB C2H2 zinc finger protein thought to function as a growth hormone-dependent transcription factor. Unfortunately, the biological function of *ZNF292* is still unclear. Both patients with *ZNF292* LGD DNMs meet diagnostic criteria for ASD (DSM-IV). Besides autism-related phenotypes, both showed delayed language development and abnormal EEG patterns. Patient M02463 (p.S832Ifs*28) showed mild ID and attention deficit hyperactivity disorder; however, patient M32023 (p.S1473Ffs*5) presented with normal IQ (108). Besides the three LGD DNMs reported in ASD patients, there are two LGD DNMs reported in DD and ID patients [24, 25]. Despite this excess of DNMs, it should be noted that LGD mutations have been reported in ExAC; unfortunately, the phenotype of these individuals cannot be further assessed.

Although not yet significant, *RALGAPB* is also a promising risk gene for follow-up as recurrent LGD DNMs were identified in ASD. In addition, an LGD DNM was also identified in a patient with epilepsy. *RALGAPB* encodes a Ras-like GTPase-activating protein. Several genes encoding the GTPase-activating protein have been associated with autism risk, such as *SYNGAP1*, *TSC2*, *ARHGAP32*, and *ARHGAP33* [17, 32, 33]. Of note, dysregulation of the Ras signaling pathway is a well-known etiologic factor from both genetic and functional studies associated with autism [34, 35].

*CHD8* is a well-described high-impact ASD gene with no LGD mutation identified in well-defined controls [13]. LGD mutations are estimated to be extremely rare in the general population, such as ExAC (minor allele frequency = 5 × 10^−5^). Here, we describe five families with inherited LGD or CNV events. Parent carriers possess mild neurodevelopmental phenotypes, including borderline IQ and broader autism phenotypes suggesting variable expressivity as opposed to a non-penetrant mutation with no phenotypic consequence. One possibility for this variability may be that a heterozygous loss-of-function mutation can cause a mild phenotype but is, by itself, not necessary and sufficient to result in an autism diagnosis unless it occurs in conjunction with other risk mutation(s). Alternatively, carrier parents who were not previously diagnosed with a neurodevelopmental disorder may harbor protective genetic variants that dampen a more severe clinical presentation of ASD. From a clinical perspective, the two are difficult to discern, but in either scenario, early diagnosis and family counseling are particularly important.

Consistent with this observation, our data also indicate that multiple DNMs in different autism risk genes within the same patient play an important role in both ASD etiology as well as disease severity. Although previous studies have shown that DNMs affect a continuum of functional outcomes [36], we investigated broader phenotypes, including occurrence of seizures. The observed association between severity of autism symptomatology and number of DNMs may provide some mechanistic insight into the heterogeneity of impairments in ASD. Such oligogenic effects have been observed previously for large CNVs associated with DD [37] and have been noted in several recent studies of ASD [38–40]. The model is distinct from a polygenic one because it puts forward that a relatively small number of rare or DNMs of large effect are primarily responsible for disease etiology and phenotypic severity, although the outcome may still be influenced by other factors, such as common variants, environment, or stochasticity during development. The analysis of SSC whole-exome sequencing data also reveals that, compared to male samples, females demonstrated an increased odds ratio for additional DNMs, although it should be noted that this study was limited to a relatively small number individuals where only exonic mutations were detected. Nevertheless, this result is consistent with the “female protective model,” which has been proposed with both genetic and epidemiological evidence in ASD [11, 29]. If this multifactorial model and female protective effect are more broadly applicable, the increased sensitivity afforded by whole-genome sequence may become more important than targeted approaches, such as exome or molecular inversion probe (MIP) sequencing, for diagnosis, discovery, and understanding of the genetic architecture and sex bias of ASD.

## Conclusions

Targeted sequencing of candidate genes in the ACGC has identified novel ASD risk genes, mutations, and genotype–phenotype relationships. Among well-established autism risk genes primarily associated with DNMs, we identify ASD families where deleterious mutations are transmitted and find that parental carriers most often show a subset of milder phenotypes. We also identify families where patients carried DNMs in two or more autism risk genes and such individuals appear to be more severely affected. Both observations provide further support for a multifactorial model of ASD risk and suggest that a monogenic model of disease will be too simplistic even for the most penetrant causes of ASD.

## Additional files

Additional file 1: **Figure S1.** Location distribution of clinical centers in the Autism Clinical and Genetic Resources in China (ACGC). (PDF 1173 kb)

Additional file 2: **Data 1.** QC for samples and genes. (XLSX 141 kb)

Additional file 3: **Figure S2.** QC of MIPs cohort. QC analysis of the percentage of MIPs with at least eight reads per sample. (PDF 84 kb)

Additional file 4: **Figure S3.** Fraction of target based on > 8-fold sequence coverage by gene. Box and whisker plots show the fraction of a sample’s target bases at 8X or greater coverage split by gene. All capture samples are included (along with QC failures). (PDF 109 kb)

Additional file 5: **Data 2.** Validation results for LGD and MIS30+ variants. (XLSX 105 kb)

Additional file 6: **Data 3.** Validation results for MIS30- variants. (XLSX 139 kb)

Additional file 7: **Data 4.** Summary of DNMs detected in this study. (XLSX 25 kb)

Additional file 8: **Figure S4.** Distribution of DNMs for *SCN2A* and *CHD8*. LGD (red) and missense (blue) DNMs with respect to the protein model in the ACGC cohort (above the protein model) are compared to previously published DNMs (below the model) primarily from European cohorts. †DNMs unique to Phase II samples; *DNMs from SSC and ASC cohorts. (PDF 931 kb)

Additional file 9: **Figure S5.** Distribution of missense mutations in *SCN2A* and *CHD8*. a. Missense DNMs in *SCN2A* are mainly located in the ion transport domain. Three recurrent missense DNM sites were identified at R937 (4), R379 (2), and G1744 (2). b. Distribution of missense DNMs in *CHD8*. One recurrent missense DNM site was identified at M904. c. The overall CADD score distributions of the missense DNMs within *SCN2A* and *CHD8* are significantly higher than the distribution of rare missense mutations of *SCN2A* and *CHD8* from ExAC. *P* values were corrected for the two tests. (PDF 1001 kb)

Additional file 10: **Figure S6.** Distribution of mutations in some of the top mutated genes (*DYRK1A*, *ASXL3*, *WDFY3* and *MECP2*) in the ACGC cohort (above) compared to previously published LGD and missense DNMs identified in the SSC and ASC cohorts. (PDF 896 kb)

Additional file 11: **Data 5.** Summary of clinical information for some patients with DNMs. (XLSX 19 kb)

Additional file 12: **Figure S7.** Logistic regression model performed to test the relationship between affected probability and DNM numbers correcting for father’s age at birth and gender. The logistic regression histogram plot shows that individuals with more DNM numbers are more likely to be affected. (PDF 879 kb)

Additional file 13: **Figure S8.** Multiple-hit model for ASD excluding DNM cases. Shown are comparisons of autism probands and unaffected siblings with one or more DNM. Logistic histograms compare residual DNM counts (DNM number residuals, note: after correction, a residual of 0 does not represent a count of 0) adjusted for the father’s age at birth and gender, and the probability of being a proband or unaffected sibling. This analysis demonstrates an increased burden of multiple hits among affected individuals (OR = 1.15, *p* = 0.0278) (a). When samples are stratified by genetic sex, we observe a slight increase but no significant effect among males (OR = 1.09, *p* = 0.6) (b), while females demonstrate a stronger (OR = 1.3, *p* = 0.037) (c) effect than the grouped analysis. (PDF 957 kb)

Additional file 14: **Figure S9.** Parent carriers of *CHD8* LGD mutations show autistic traits. The density plots are based on the BAPQ scores of all SSC parents. Left: father; Right: mother. Red arrows point to the corresponding BAPQ scores of the three parents with *CHD8* LGD mutations. (PDF 249 kb)

## Abbreviations

AC: Allele count
ACGC: Autism Clinical and Genetic Resources in China
ASC: Autism Sequencing Consortium
ASD: Autism spectrum disorders
BAPQ: Broad Autism Phenotype Questionnaire
CADD: Combined Annotation Dependent Depletion
CH model: chimpanzee-human divergence model
CNV: Copy number variant
DD: developmental delay
DN: De novo
DNM: De novo mutation
FDR: False discovery rate
HC: Head circumference
ID: intellectual disability
LGD: Likely gene-disrupting
NVIQ: Nonverbal intelligence quotient
OR: Odds ratio
QC: Quality control
SFARI: Simons Foundation Autism Research Initiative
smMIPs: single-molecule molecular inversion probes
SRS: Social Responsiveness Scale
SSC: Simons Simplex Collection
