# Supplementary material for: Inherited and multiple de novo mutations in autism/developmental delay risk genes suggest a multifactorial model
Source: Mol Autism. 2018 Dec 13;9:64. doi: 10.1186/s13229-018-0247-z (PMC6293633; doi:10.1186/s13229-018-0247-z)
Supplement: Supplementary file 1 — Figure S1. Location distribution of clinical centers in the Autism Clinical and Genetic Resources in China (ACGC). (PDF 1173 kb) [file 13229_2018_247_MOESM1_ESM.pdf]

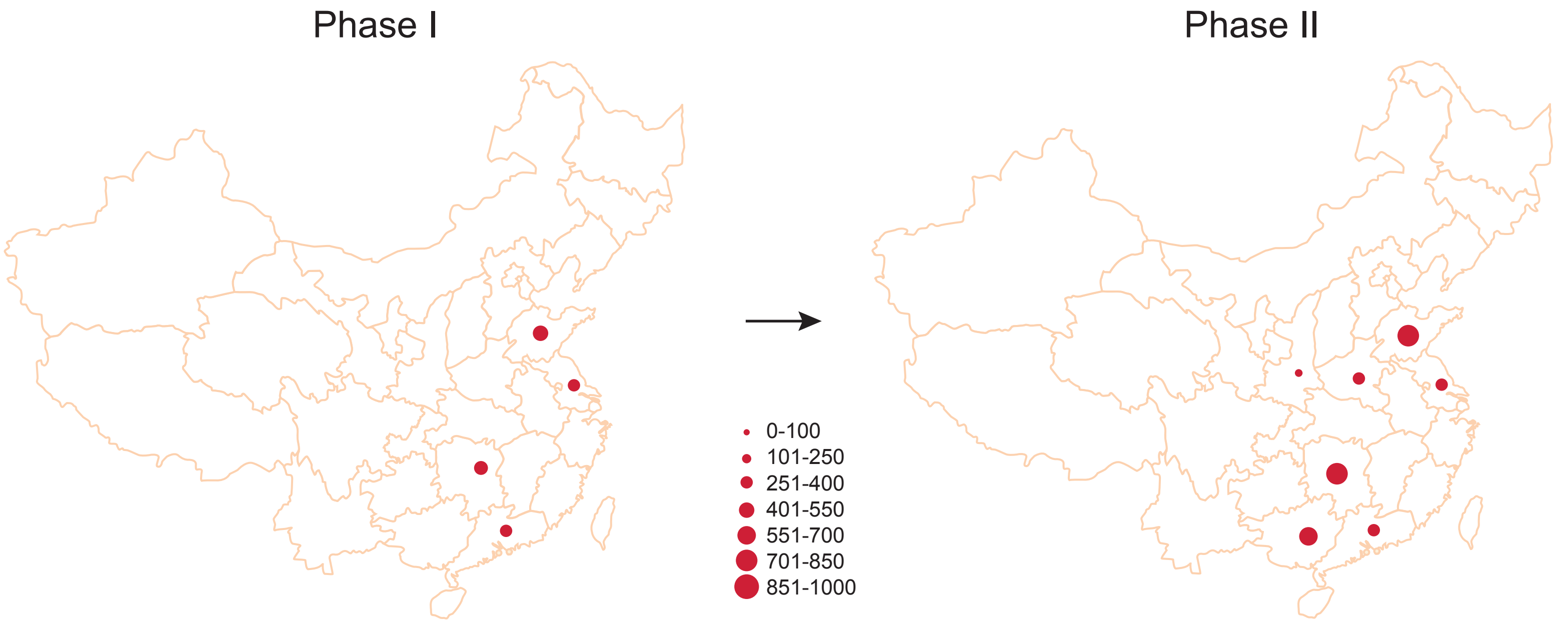

Hunan:	Mental Health Institute, the Second Xiangya Hospital, Central South University, Changsha, Hunan, China
Guangdong:	Children’s Development Behavior Center, Third Affiliated Hospital of Sun Yat-sen University, Guangzhou, Guangdong, China
Shandong:	Mental Health Center of Shandong Province, Jinan, Shandong, China
Jiangsu:	Child Mental Health Research Center, Nanjing Brain Hospital Affiliated of Nanjing Medical University, Nanjing, Jiangsu, China
Guangxi:	Child Healthcare Department, Liuzhou Maternity and Child Healthcare Hospital, Liuzhou, Guangxi, China
Henan:	Child Healthcare Department, The Third affiliated hospital of Zhengzhou University, Zhengzhou, Henan China
Shaanxi:	Xi 'an Encephalopathy Hospital of Traditional Chinese Medicine, Xi 'an, Shaanxi, China
