# Supplementary figures and images for: Inherited and multiple de novo mutations in autism/developmental delay risk genes suggest a multifactorial model
Source: Mol Autism. 2018 Dec 13;9:64. doi: 10.1186/s13229-018-0247-z (PMC6293633; doi:10.1186/s13229-018-0247-z)

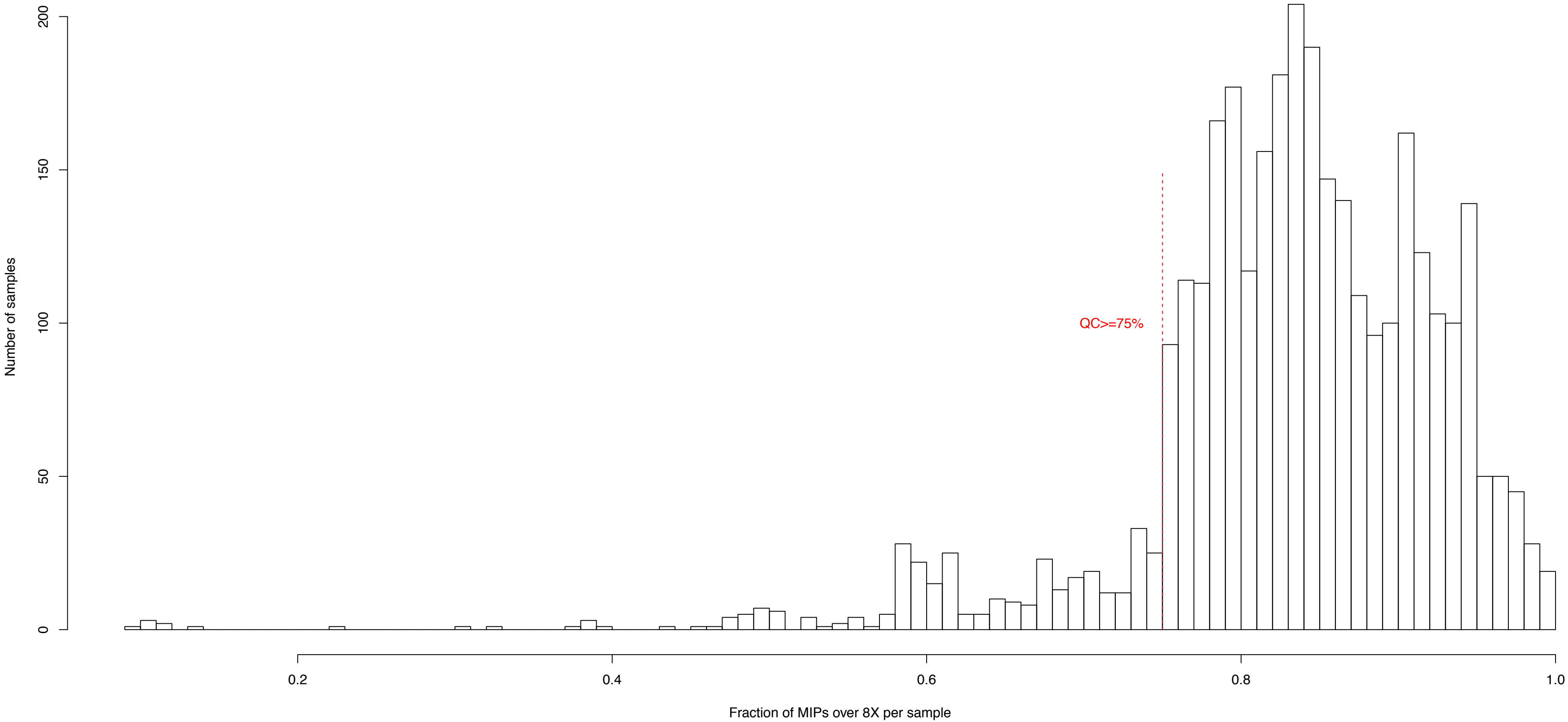

Supplement: Supplementary file 3 — Figure S2. QC of MIPs cohort. QC analysis of the percentage of MIPs with at least eight reads per sample. (PDF 84 kb) [file 13229_2018_247_MOESM3_ESM.pdf]

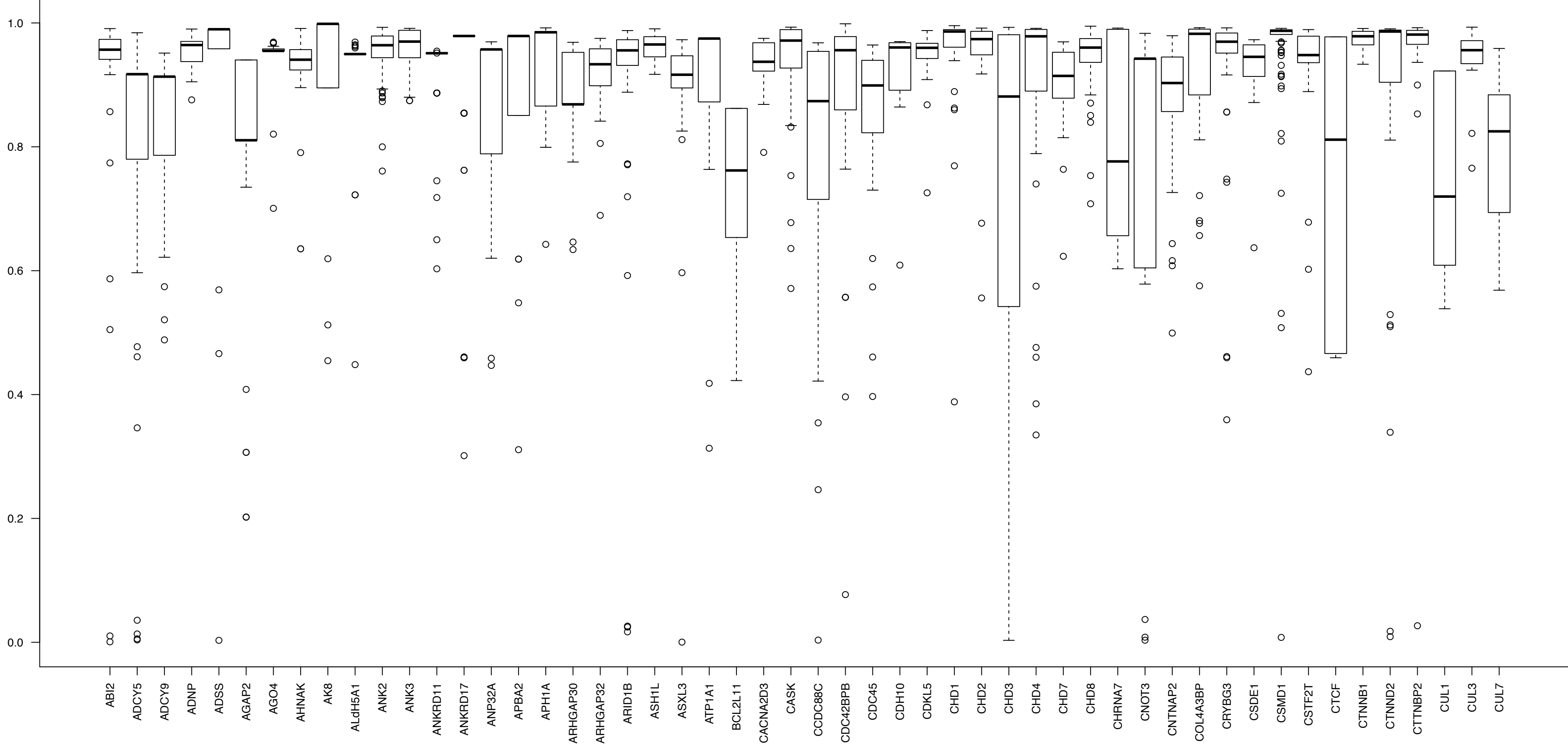

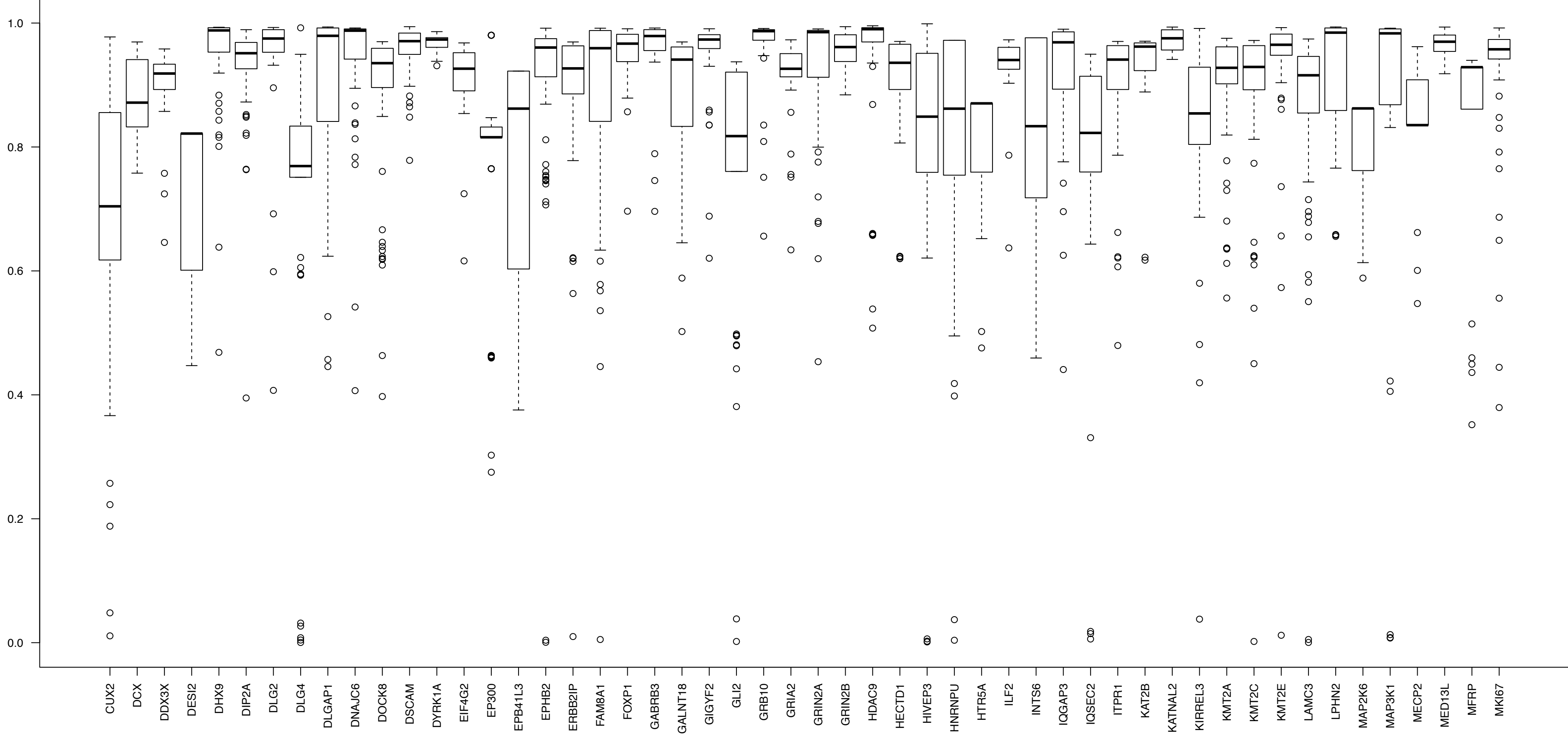

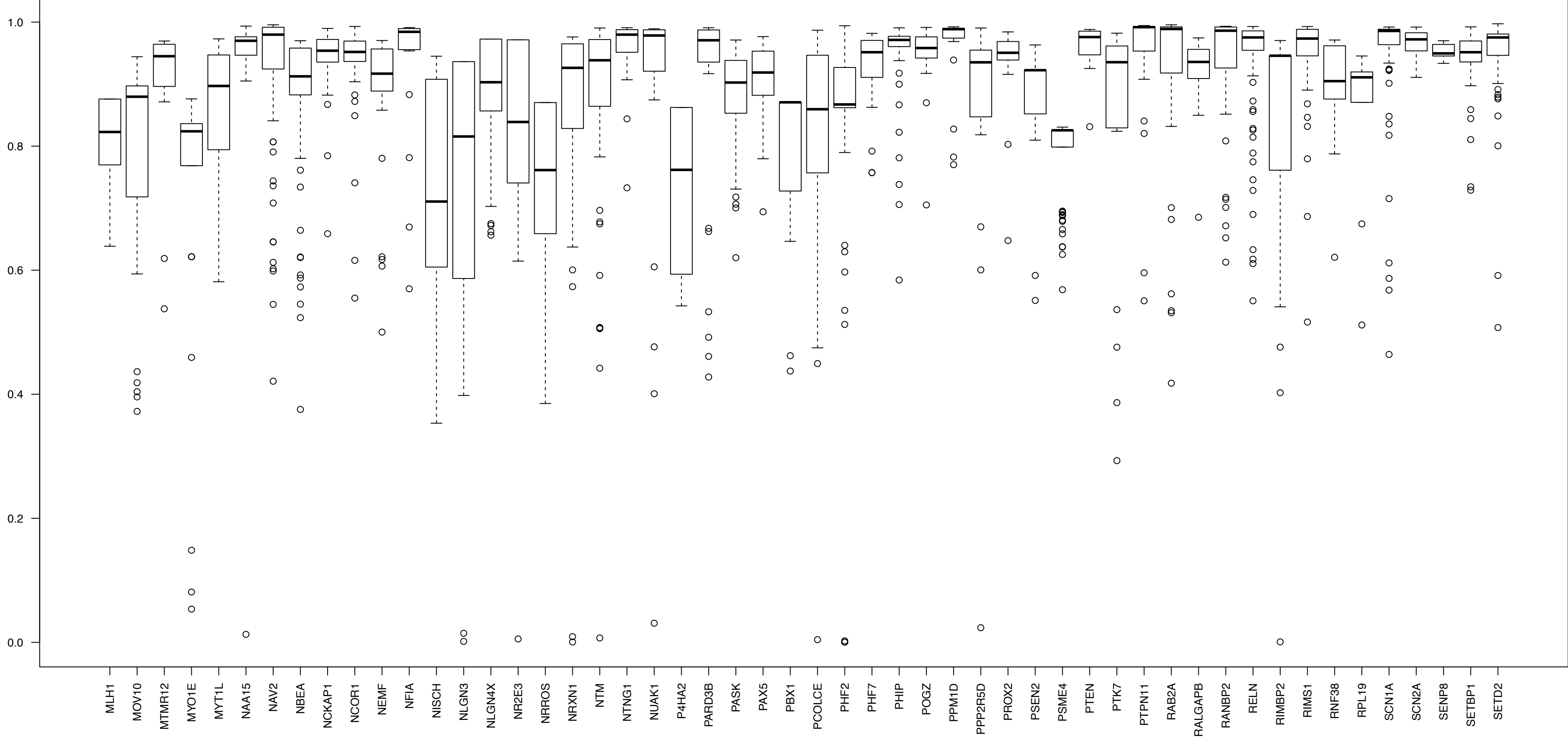

Fraction of samples

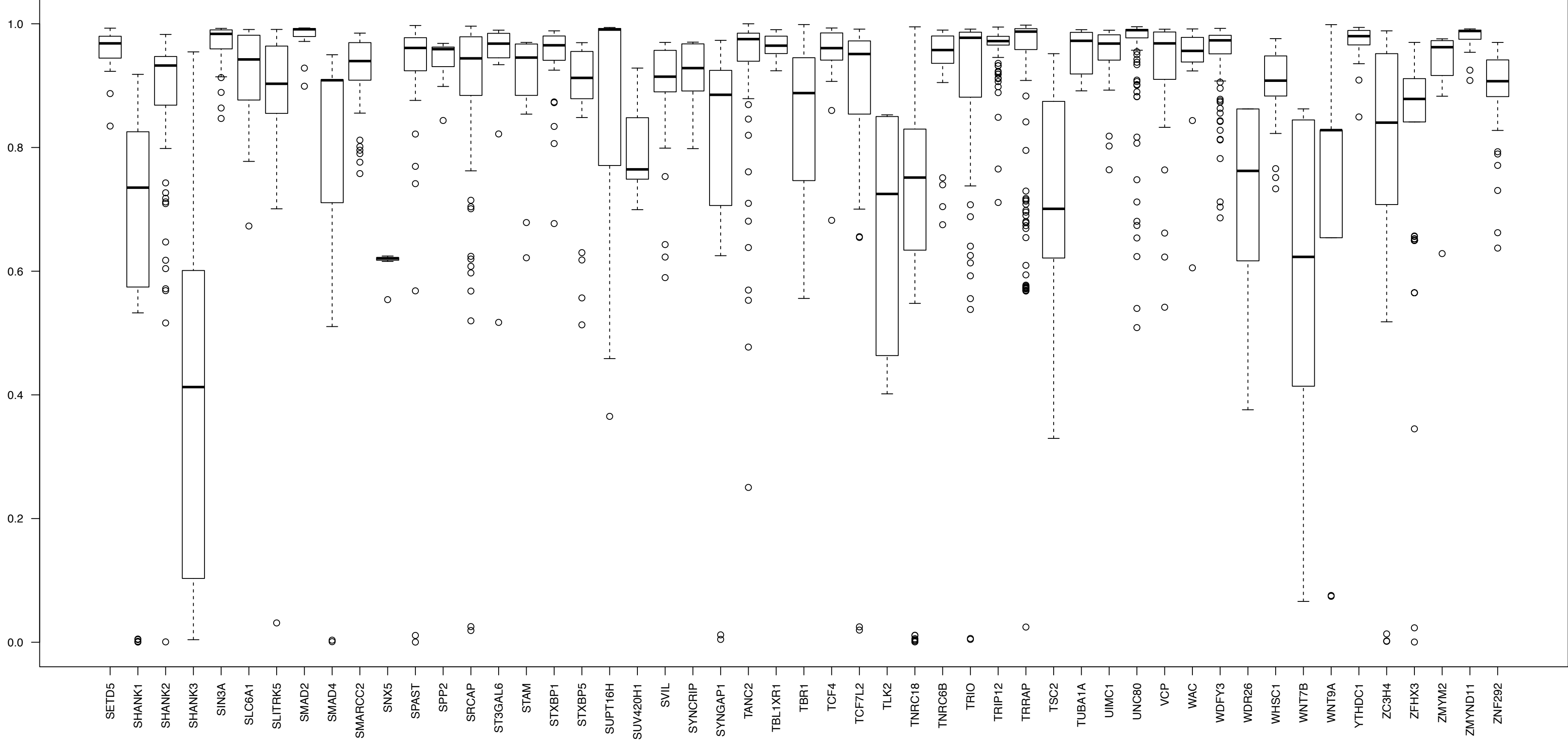

Supplement: Supplementary file 4 — Figure S3. Fraction of target based on > 8-fold sequence coverage by gene. Box and whisker plots show the fraction of a sample’s target bases at 8X or greater coverage split by gene. All capture samples are included (along with QC failures). (PDF 109 kb) [file 13229_2018_247_MOESM4_ESM.pdf]

NM\_001040142.1 → NP\_001035232.1

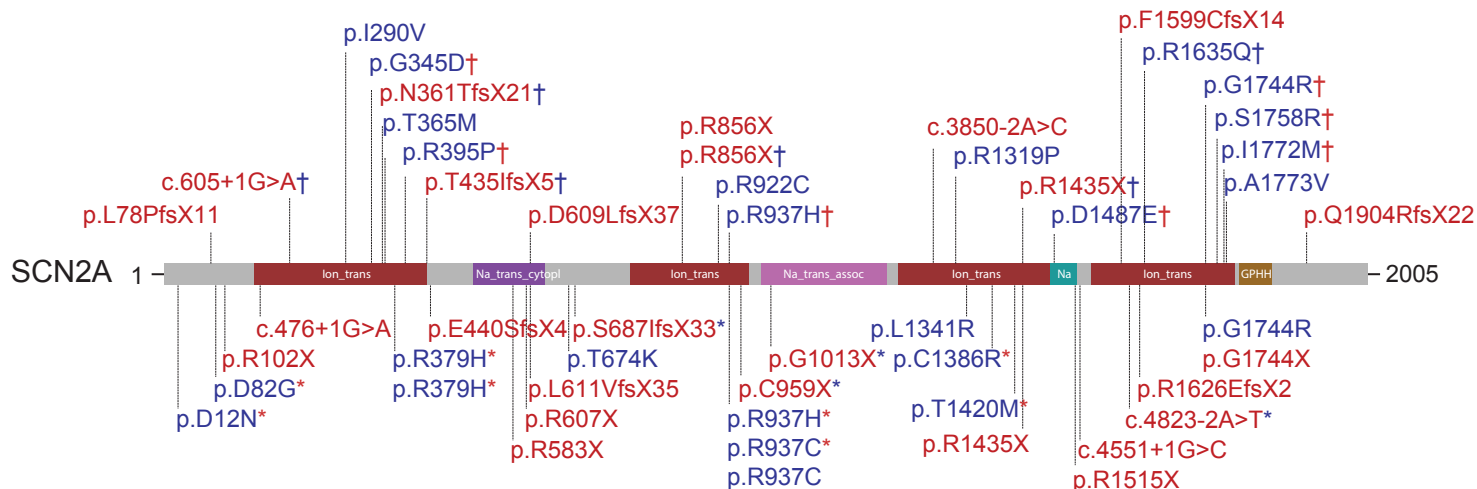

NM\_001170629.1 → NP\_001164100.1

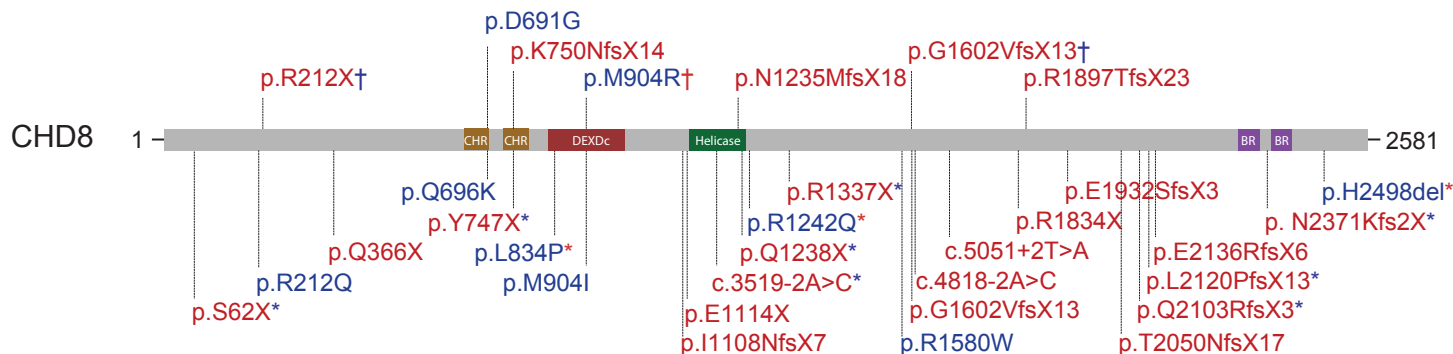

Supplement: Supplementary file 8 — Figure S4. Distribution of DNMs for SCN2A and CHD8. LGD (red) and missense (blue) DNMs with respect to the protein model in the ACGC cohort (above the protein model) are compared to previously published DNMs (below the model) primarily from European cohorts. †DNMs unique to Phase II samples; *DNMs from SSC and ASC cohorts. (PDF 931 kb) [file 13229_2018_247_MOESM8_ESM.pdf]

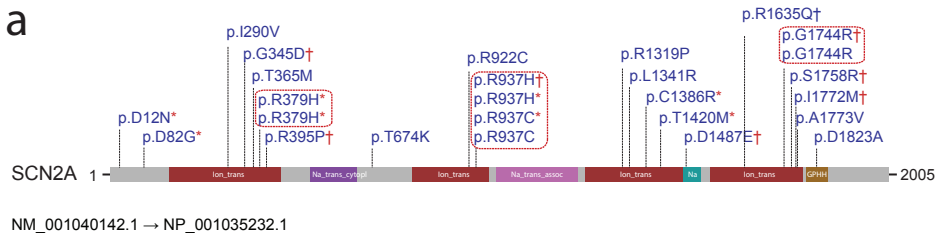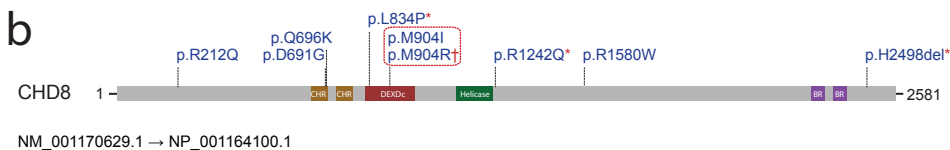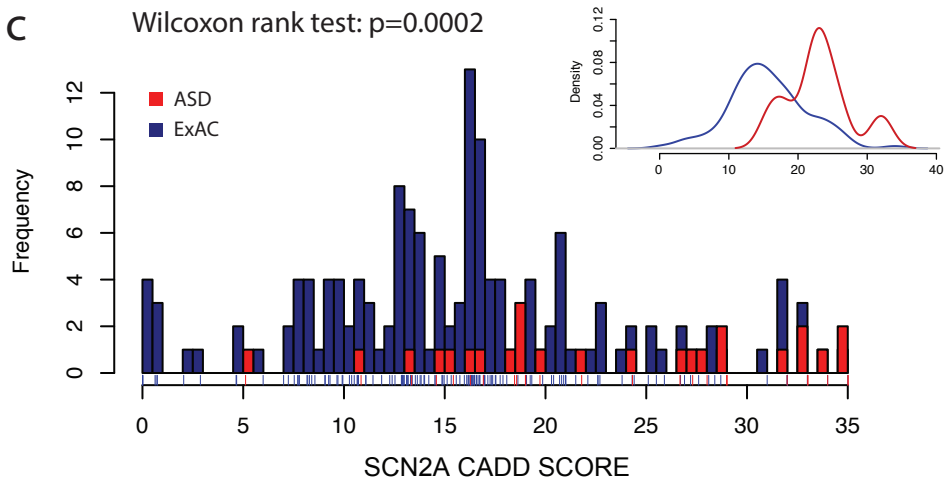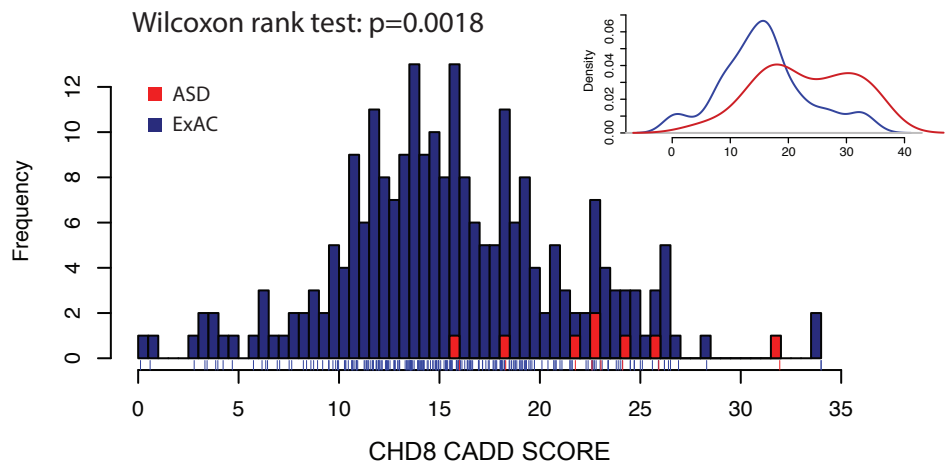

Supplement: Supplementary file 9 — Figure S5. Distribution of missense mutations in SCN2A and CHD8. a. Missense DNMs in SCN2A are mainly located in the ion transport domain. Three recurrent missense DNM sites were identified at R937 (4), R379 (2), and G1744 (2). b. Distribution of missense DNMs in CHD8. One recurrent missense DNM site was identified at M904. c. The overall CADD score distributions of the missense DNMs within SCN2A and CHD8 are significantly higher than the distribution of rare missense mutations of SCN2A and CHD8 from ExAC. P values were corrected for the two tests. (PDF 1001 kb) [file 13229_2018_247_MOESM9_ESM.pdf]

NM\_001396.4 → NP\_001387.2

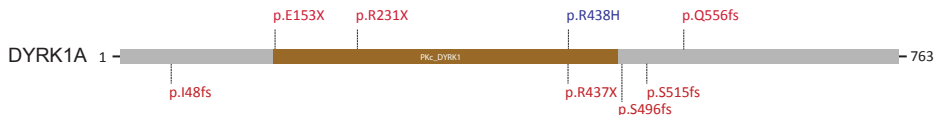

NM\_030632.2 → NP\_085135.1

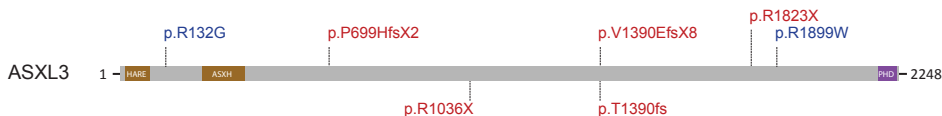

NM\_014991.4 → NP\_055806.2

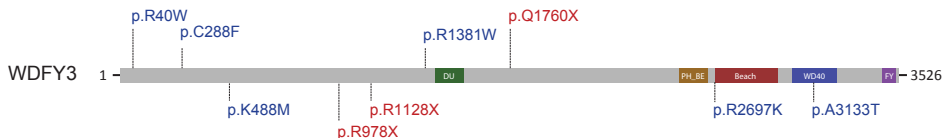

NM\_00110792.1 → NP\_001104262.1

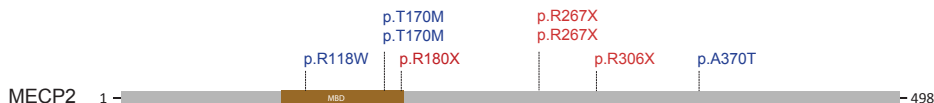

Red = LGD DNM; Blue = missense DNM

Supplement: Supplementary file 10 — Figure S6. Distribution of mutations in some of the top mutated genes (DYRK1A, ASXL3, WDFY3 and MECP2) in the ACGC cohort (above) compared to previously published LGD and missense DNMs identified in the SSC and ASC cohorts. (PDF 896 kb) [file 13229_2018_247_MOESM10_ESM.pdf]

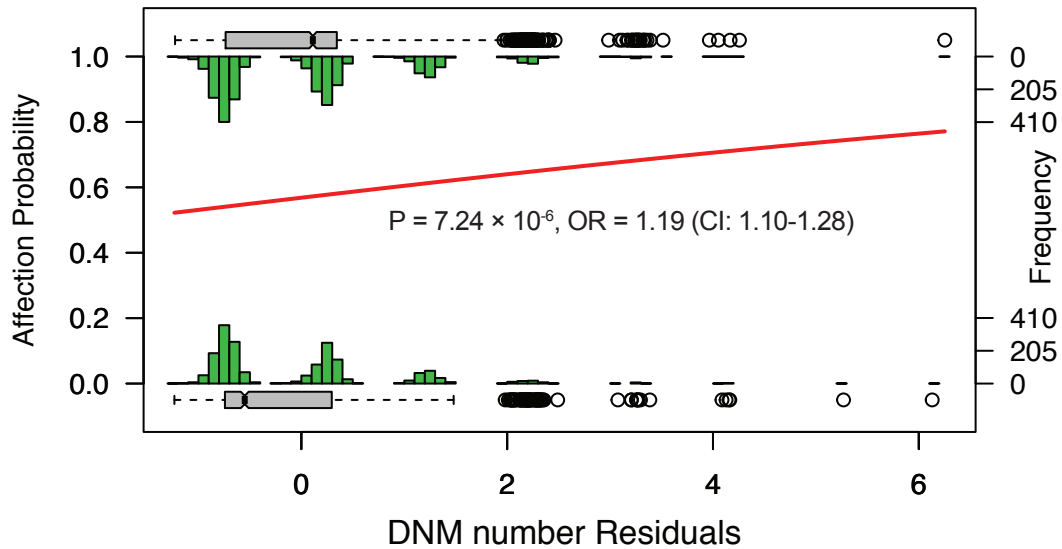

Supplement: Supplementary file 12 — Figure S7. Logistic regression model performed to test the relationship between affected probability and DNM numbers correcting for father’s age at birth and gender. The logistic regression histogram plot shows that individuals with more DNM numbers are more likely to be affected. (PDF 879 kb) [file 13229_2018_247_MOESM12_ESM.pdf]

a

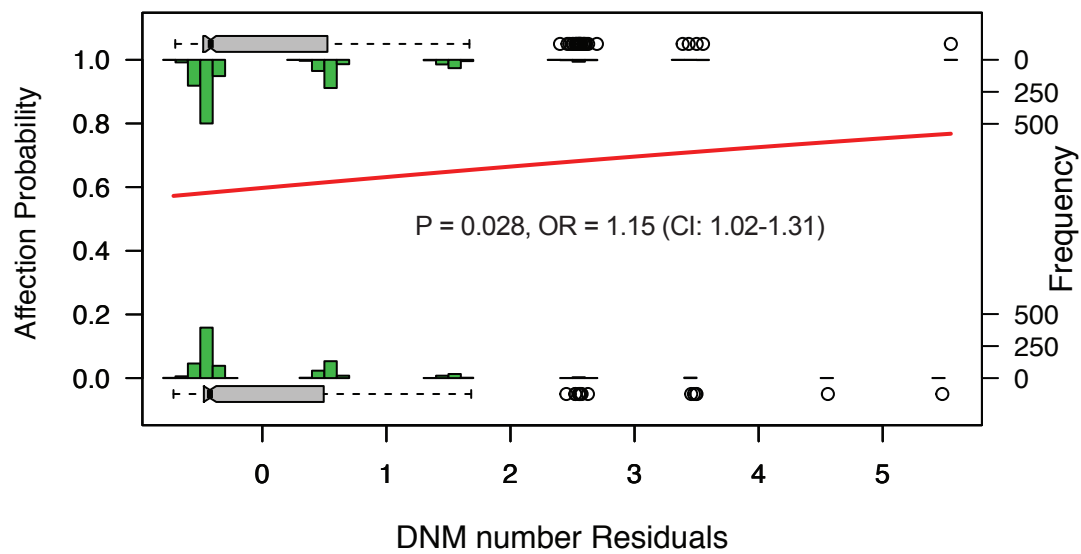

b

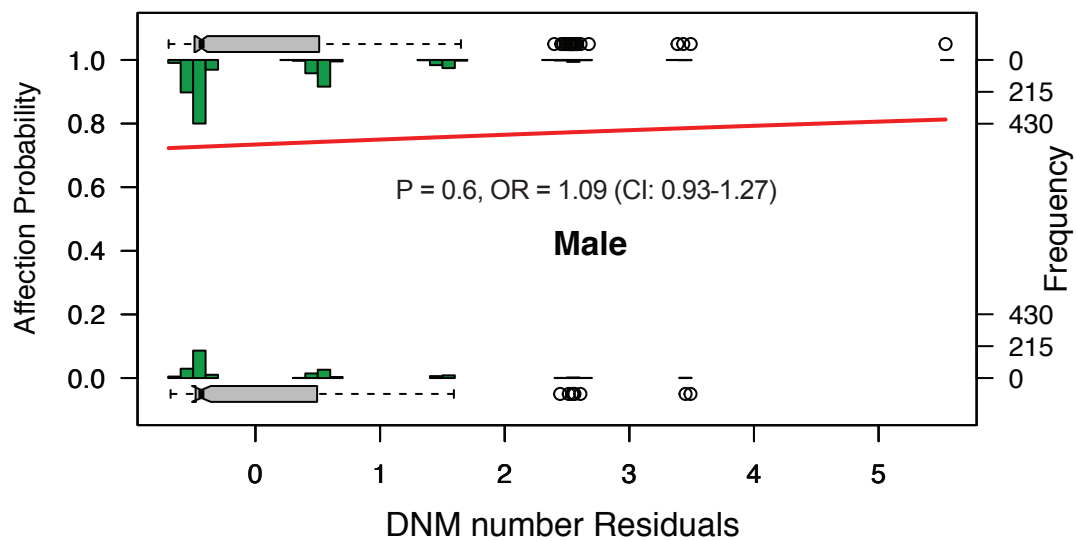

c

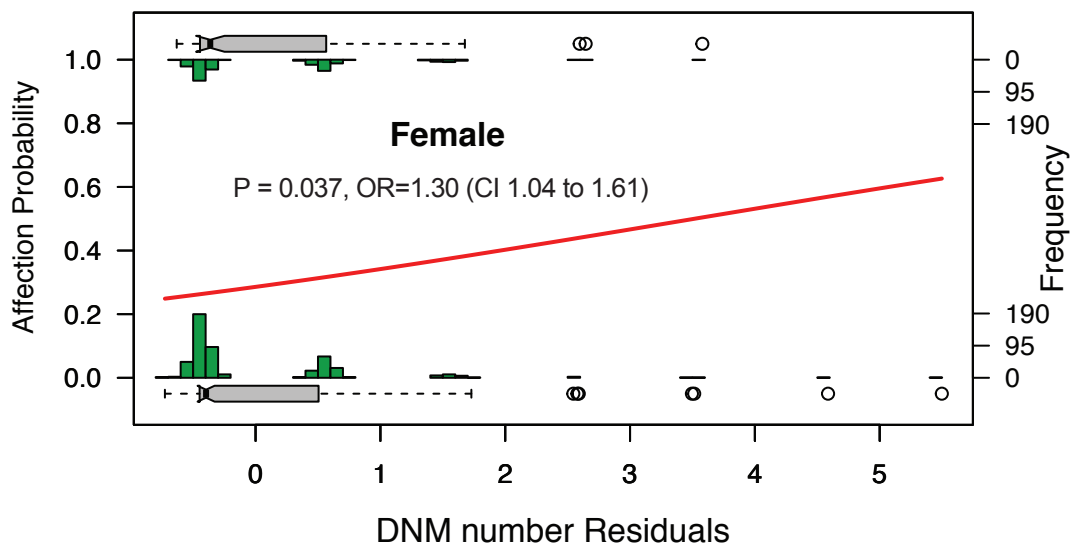

Supplement: Supplementary file 13 — Figure S8. Multiple-hit model for ASD excluding DNM cases. Shown are comparisons of autism probands and unaffected siblings with one or more DNM. Logistic histograms compare residual DNM counts (DNM number residuals, note: after correction, a residual of 0 does not represent a count of 0) adjusted for the father’s age at birth and gender, and the probability of being a proband or unaffected sibling. This analysis demonstrates an increased burden of multiple hits among affected individuals (OR = 1.15, p = 0.0278) (a). When samples are stratified by genetic sex, we observe a slight increase but no significant effect among males (OR = 1.09, p = 0.6) (b), while females demonstrate a stronger (OR = 1.3, p = 0.037) (c) effect than the grouped analysis. (PDF 957 kb) [file 13229_2018_247_MOESM13_ESM.pdf]

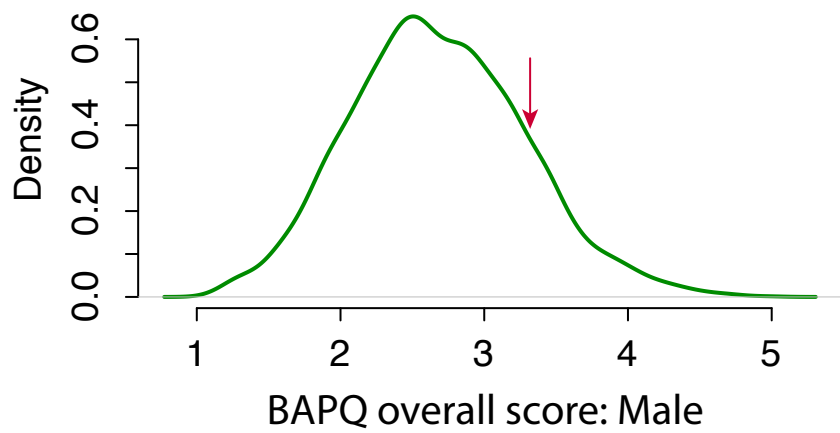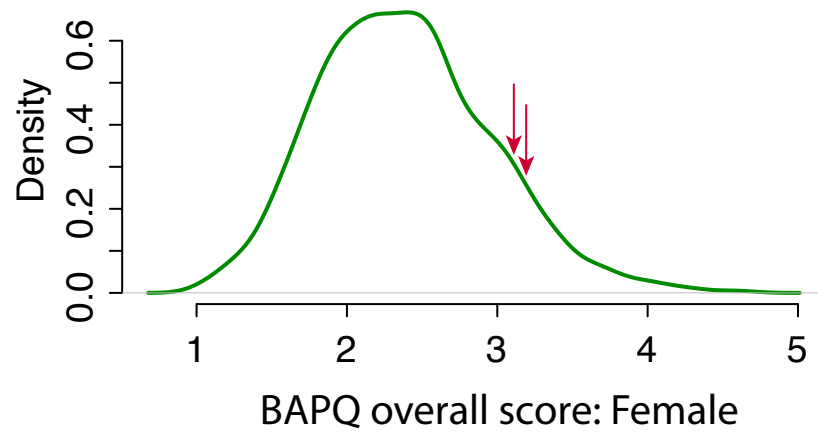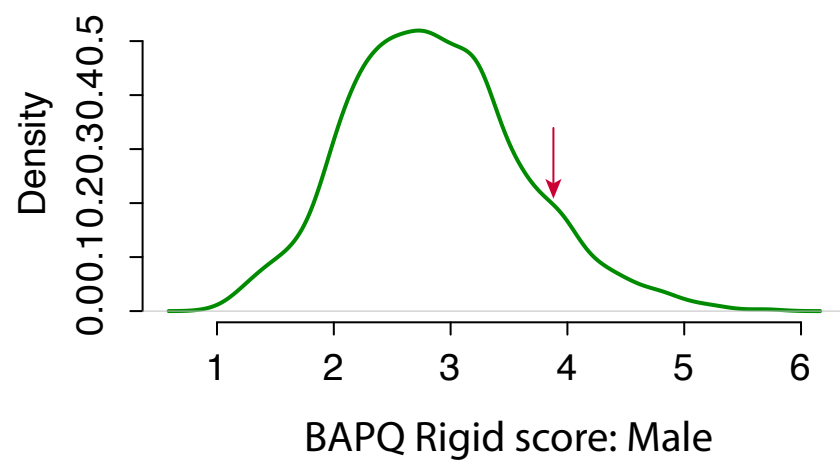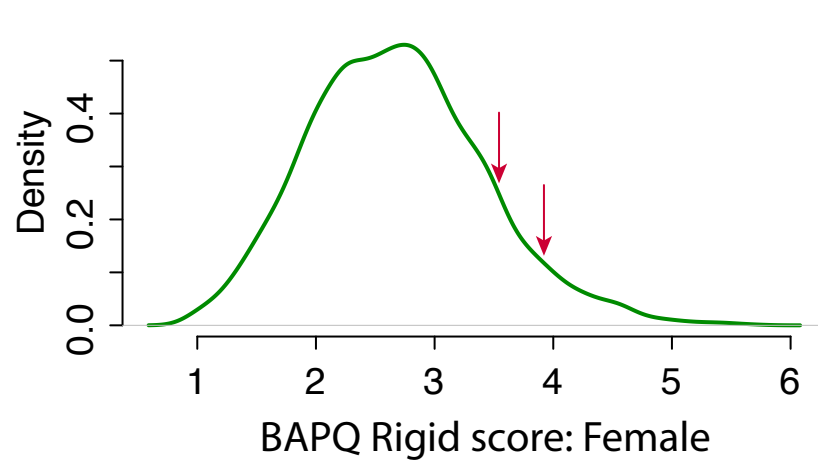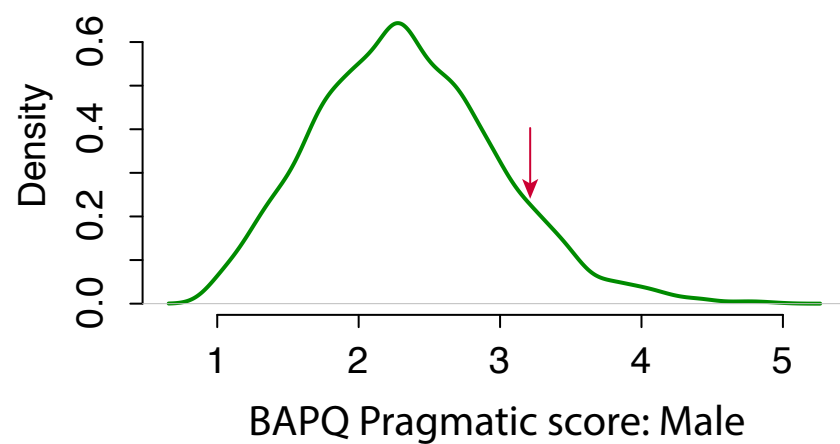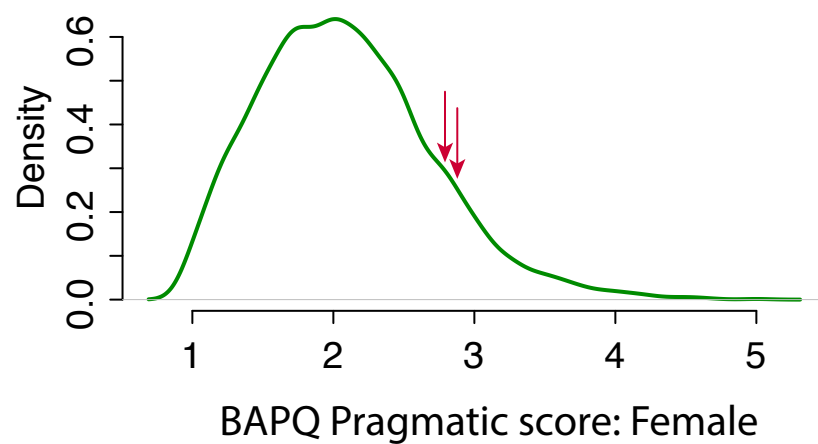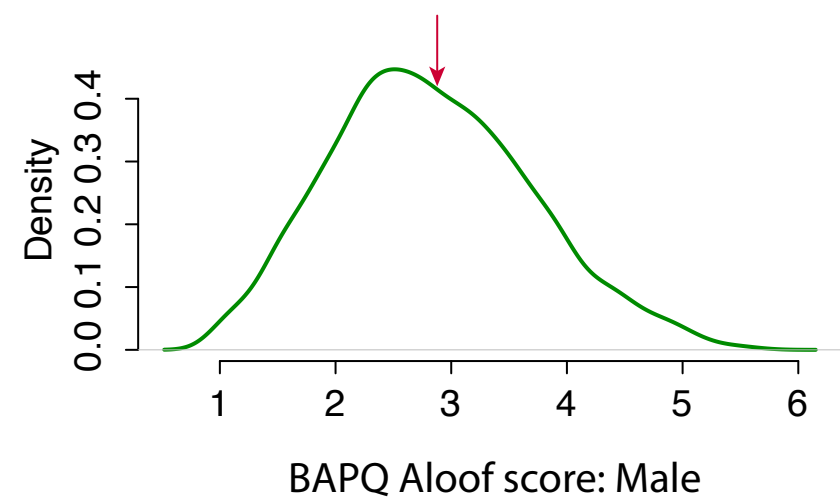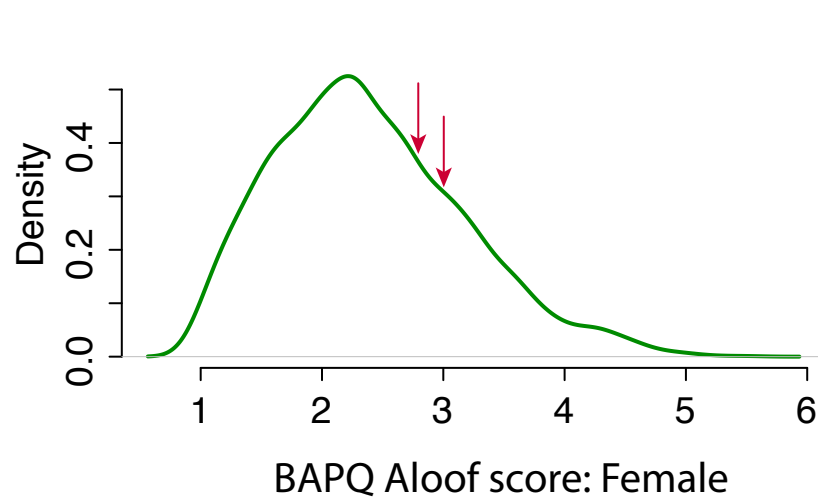

Supplement: Supplementary file 14 — Figure S9. Parent carriers of CHD8 LGD mutations show autistic traits. The density plots are based on the BAPQ scores of all SSC parents. Left: father; Right: mother. Red arrows point to the corresponding BAPQ scores of the three parents with CHD8 LGD mutations. (PDF 249 kb) [file 13229_2018_247_MOESM14_ESM.pdf]
